# Artificial intelligence in colorectal cancer multidisciplinary decision-making: concordance, predictive support and clinical translation

**DOI:** 10.3389/fonc.2026.1909120

**Published:** 2026-09-14

**Authors:** Aristotelis Nikitaras, Sandra Maria Tsoti, Manousos-Georgios Pramateftakis

**Affiliations:** 1 Athens Euroclinic, Athens, Greece; 2 Saint Savvas General Anticancer-Oncology Hospital of Athens, Athens, Greece; 3 Aristotle University of Thessaloniki, Thessaloniki, Greece

**Keywords:** artificial intelligence, clinical decision support, colorectal cancer, multidisciplinary team, precision oncology, treatment planning

## Abstract

Multidisciplinary teams (MDTs) are central to colorectal cancer management, where treatment decisions increasingly depend on the integration of tumor stage, molecular characteristics, patient fitness, and multimodal treatment strategies. However, MDT workflows are time-consuming, subject to inter-team variability, and influenced by differences in expertise, local practices, and resource availability. Artificial intelligence (AI) has emerged as a potential support tool for data integration, standardization, and risk stratification. This review examines AI in colorectal cancer multidisciplinary decision-making, focusing on AI–MDT concordance, predictive models with potential relevance to MDT discussions, and clinical translation. Reported concordance between large language models and MDT decisions varies substantially and appears to be influenced by disease context, age, performance status, case complexity, input quality, and the inclusion of clinically important variables. Predictive models may provide additional prognostic information relevant to treatment planning. Prospective evidence of AI integrated into colorectal cancer MDT workflows remains very limited. Nevertheless, the literature remains limited by predominantly retrospective designs, small or selected cohorts, heterogeneous endpoints, and unresolved issues related to transparency, reproducibility, regulation, and accountability. Current evidence is insufficient to establish improvements in MDT decision quality or patient outcomes, and AI should therefore be regarded as a supervised support tool rather than a replacement for expert multidisciplinary judgment.

## 1 Introduction

Colorectal cancer (CRC) is the third most common cancer and the second leading cause of cancer-related death worldwide (1, 2). Treatment decisions depend on tumor stage, molecular characteristics, patient fitness, and multimodal strategies including surgery, systemic therapy, radiotherapy, and surveillance (3–6). Consequently, colorectal cancer decision-making requires multidisciplinary coordination and interpretation of diverse clinical information to select the most appropriate treatment plan (7).

Multidisciplinary teams (MDTs) are fundamental to colorectal cancer decision-making. By bringing together surgeons, medical oncologists, radiation oncologists, radiologists, and pathologists, MDTs enable comprehensive case evaluation and individualized treatment (8). Their use has been associated with improved guideline adherence, treatment selection, and clinical outcomes (8, 9). However, MDT decision-making is time-consuming, depends on specialist availability and expertise, and may vary with interpretation and institutional practice (8). Increasing treatment complexity places additional pressure on MDT workflows and highlights the need for tools to facilitate and standardize decision-making (10).

Artificial intelligence (AI) has emerged as a potential tool in oncology, with applications ranging from imaging analysis and endoscopic assistance to risk prediction and clinical decision support (11–17). By integrating complex datasets, AI may help clinicians synthesize information and support more consistent decision-making. In MDT discussions, AI-based systems may improve efficiency, support guideline adherence, reduce variability, and assist interpretation of clinical data (18, 19). However, clinical integration into MDT workflows remains limited, and concerns regarding reliability, interpretability, and validation persist (19, 20). This Mini Review evaluates current evidence on AI in colorectal cancer multidisciplinary decision-making, focusing on clinical applications, limitations, and future perspectives.

Recent systematic reviews address complementary aspects of this field. Tian et al. reviewed large language model (LLM) applications across the colorectal cancer care continuum, including treatment decision support (21); Konzmann et al. focused on LLMs in tumor boards across various cancers (22); and Wu et al. evaluated broader AI decision support systems in surgical oncology MDTs, including colorectal applications (23). Within this broader literature, this Mini Review provides a focused narrative synthesis of colorectal cancer multidisciplinary decision-making, drawing on contemporary LLM and relevant non-LLM AI evidence. It organizes the discussion around AI–MDT concordance, predictive or prognostic support, and clinical translation, while distinguishing direct MDT evidence from evidence with indirect relevance to future MDT integration. It also distinguishes MDT concordance from independent guideline concordance and from demonstrated effects on MDT decisions, workflow, or patient outcomes.

A targeted PubMed search was conducted on 1 April 2026 using (“colorectal cancer”) AND (“artificial intelligence”) AND (“decision support” OR “multidisciplinary team” OR “tumor board”), limited to English-language human studies from the preceding five years. Studies directly relevant to treatment or multidisciplinary decision-making were prioritized; predictive models and clinically implemented decision-support studies without direct MDT evaluation were considered separately where relevant to potential translation into multidisciplinary care. Preprints were excluded from the final synthesis and mixed-cancer evidence was included selectively when relevant. Supplementary targeted searches during peer review addressed disease stage, clinical complexity, explainable AI, newly published or accepted studies, and representative earlier non-LLM decision-support systems.

## 2 Challenges of MDT decision-making in colorectal cancer

In routine practice, MDTs discuss high case volumes within limited time and organizational constraints (24). Clinical, radiological, and pathological information may also be incomplete or of variable quality at the time of discussion. Time pressure may limit the depth of analysis, particularly in high-volume centers, while the need for consensus may favor streamlined over fully individualized recommendations. MDT decisions are also influenced by team composition, experience, local resources, and institutional protocols (8, 24, 25).

Another significant limitation of MDT decision-making is the variability between teams and between institutions. Differences in clinical experience, interpretation of imaging or pathology findings, and familiarity with evolving evidence may lead to inconsistent recommendations, especially in complex or borderline cases (8). Additionally, decision-making may be influenced by local practices, the availability of technology, and access to specific treatments, which can affect the uniform application of guidelines (24). Together, these factors make consistent and standardized colorectal cancer management more difficult.

## 3 Current evidence relevant to AI in MDT decision-making

### 3.1 Concordance between AI and MDT recommendations

The evidence base on AI–MDT concordance in colorectal cancer ranges from earlier non-LLM decision-support systems to more recent LLM-based approaches. Among the earlier non-LLM approaches, colorectal cancer-specific decision-tree systems included OncoSurge and Oncoguide, which were evaluated against MDT recommendations, while other knowledge-based approaches have also been investigated in surgical oncology (23). A later prominent non-LLM decision-support system, Watson for Oncology, was subsequently evaluated in colorectal cancer cohorts, with reported MDT concordance of 66.9% and 91% (26, 27), illustrating variable agreement across studies. These systems differ substantially from contemporary generative AI, so their concordance estimates are not directly comparable.

Recent direct evidence is derived mainly from retrospective, single-center studies comparing LLM-generated management recommendations with expert colorectal MDT decisions. These studies evaluated heterogeneous clinical populations, ranging from localized disease to metastatic, recurrent, and surgically complex cases, and used different model versions, prompting strategies, treatment categories, and concordance measures. Reported agreement therefore varies substantially, and cross-study numerical comparisons should be interpreted cautiously (28–36).

A large study evaluated 1,423 colorectal cancer MDT cases using three contemporary LLMs and reported agreement rates of 62.5–65.4%, with κ values indicating fair agreement (28). Among cases with stable outputs from the best-performing model, metastatic disease was independently associated with lower concordance, whereas cT3–4 and cN-positive status were not significant predictors. However, the primary analysis excluded cases with variable outputs, which were more frequent in metastatic disease or cases with incomplete staging, and cT or cN status was undocumented in approximately half the cohort (28). In another study of 300 patients, a Gemini model provided with external guideline information achieved high agreement with tumor board decisions, although unequal external guideline access limited attribution of performance differences to intrinsic model capability (29). Exploratory subgroup analyses showed greater discordance with Gemini in stage IV and recurrent disease, whereas MedGemma demonstrated a different pattern, including greater discordance in stage II disease, patients with ECOG performance status ≥2, and recurrent disease (29). Earlier evidence from 157 patients similarly showed descriptively lower κ estimates in stage IV than in stage I–III disease in both preoperative and postoperative settings, although no formal comparison between stage groups was reported (30). Taken together, these studies suggest that advanced or recurrent disease may present greater difficulties for LLM-based decision support, but they do not demonstrate a uniform stage-dependent effect.

Smaller studies provide additional information about the influence of patient characteristics, clinical context, and input completeness. In a cohort of 100 patients, ChatGPT-4 showed complete concordance with MDT recommendations in 72.5% of pretherapeutic and 82.8% of post-therapeutic discussions. T stage was not associated with concordance. Although older age and higher ASA classification were associated with discordance in subgroup analyses, age and ASA were interdependent, and only increasing age remained independently associated in multinomial analysis (31). Concordance with the MDT does not indicate whether the AI recommendation is independently supported by clinical guidelines. In comparison with the German S3 guideline, the MDT recommendation was guideline concordant in 94.1% of substantially discordant pretherapeutic cases, while both recommendations were guideline concordant in 70% of partially discordant cases (31). In a separate cohort of 179 patients with stage II colon cancer, agreement was lower in patients aged ≥70 years, those with ECOG performance status 2, and those with multiple pathological risk factors (32). Conversely, an early study of 30 selected patients with stage IV, recurrent, or synchronous colorectal cancer reported 86.7% agreement, suggesting that advanced disease does not invariably result in poor concordance (33). Similarly, in a rectal cancer cohort encompassing all disease stages, higher concordance scores were observed in node-positive disease for two of the three models evaluated (34).

Clinical input completeness and framing also appear to be important. In 30 patients with colorectal cancer liver metastases, agreement increased from 66.7% under the baseline condition to 93.3% when resectability potential was explicitly provided as a second predefined information condition (35). This finding reflects sensitivity to the provision of a clinically decisive variable rather than an independent AI assessment of resectability. In a separate pilot study of 15 patients with anal or colorectal cancers, an LLM-based chatbot achieved a mean concordance score of 4.08/5 compared with historical MDT decisions, although interrater agreement for concordance was only moderate and agreement regarding the justification of recommendations was slight (36). Collectively, the available evidence does not support a simple linear relationship between anatomical stage and AI–MDT concordance. Metastatic or recurrent disease may reduce agreement in some settings, but age, performance status, treatment context, decision complexity, information completeness, model configuration, and institutional practice may also influence concordance.

Beyond concordance, a 20-case molecular tumor board study found that ChatGPT-4 generated more therapeutic recommendations in less time than the human tumor board, with no significant difference in the study-specific information-density metric (37). However, repeated runs showed only moderate consistency and more frequently drew on lower-level or preclinical evidence. These findings suggest potential efficiency benefits, but expert supervision remains necessary to assess the relevance and evidential basis of recommendations.

### 3.2 Predictive models with potential relevance to MDT decision-making

Beyond concordance studies, AI-based predictive models may provide prognostic information relevant to MDT decision-making. These models estimate outcomes such as recurrence or overall survival rather than generate MDT recommendations directly (16, 17). Their findings are therefore indirectly relevant to multidisciplinary decision-making rather than direct evaluations of AI-supported MDT practice.

One such study developed a web-based explainable AI clinical decision-support system to predict 5-year recurrence risk after curative colorectal cancer surgery, reporting an area under the curve of approximately 0.83–0.84 (16). The AUC reflects model discrimination but does not assess calibration or net benefit and does not establish clinical utility or decision impact. The system included an explainability component showing which clinical variables contributed to individual recurrence-risk predictions and was deployed on the hospital’s internal network with clinician training and focus-group feedback. However, this model was developed and validated retrospectively, and the effect of the explainability component on clinical decisions or patient outcomes was not prospectively assessed (16). Another retrospective study used a deep learning model based on electronic medical record data to predict 5-year overall survival and applied an explainability method to identify clinically relevant prognostic features (17). Although such models may help refine risk stratification and provide information relevant to decisions regarding adjuvant treatment intensity or follow-up, their integration into MDT workflows has not been prospectively evaluated (16, 17). Their current value therefore lies primarily in their potential to inform multidisciplinary discussions rather than in demonstrated effects on clinical decision-making or patient outcomes.

### 3.3 Clinical translation of AI-based decision support

Prospective CRC-specific evaluation of AI integrated into MDT decision-making remains very limited. In pan-oncology settings, prospective studies have evaluated AI alongside MDT decisions (38, 39) and, more recently, within live MDT deliberation, where AI input refined 10.3% and replaced 3.4% of final recommendations (39). A Danish before-and-after study illustrates broader clinical translation outside direct MDT decision-making: an AI prediction model developed using registry data from 18,403 patients undergoing colorectal cancer surgery was externally validated in 806 patients and prospectively implemented in 194 for personalized perioperative decision support (40). The model predicted 1-year mortality risk and stratified patients into four risk groups linked to predefined perioperative optimization bundles of increasing intensity. Following implementation of the AI-supported risk-stratified perioperative pathway, the personalized treatment cohort had lower rates of severe complications than the standard-care cohort (19.1% versus 28.0%), as well as fewer medical complications (23.7% versus 37.3%) and fewer readmissions. Short-term health-economic modeling suggested that the pathway was cost-effective. Although the non-randomized design does not allow firm conclusions about cause and effect, this study demonstrates that AI-based decision support can be prospectively integrated into colorectal cancer care and evaluated against clinical outcomes. However, it did not evaluate AI as decision support during MDT deliberation or its effect on MDT treatment decisions (40).

## 4 Discussion

Most contemporary CRC evidence concerns AI–MDT concordance or predictive performance, while CRC-specific prospective MDT integration remains very limited. Emerging pan-oncology studies provide prospective concordance and limited decision-impact evidence (38, 39), whereas the CRC-specific prospective example concerns AI-supported perioperative care outside direct MDT decision-making (40). These evidence types address different questions, ranging from model or decision performance to clinical and system outcomes, as illustrated qualitatively in **Figure 1**. Concordance with MDT or guideline recommendations and expert assessment of appropriateness or safety evaluate the recommendation itself, whereas decision impact, workflow effects, and patient outcomes evaluate clinical use.

**Figure 1:**
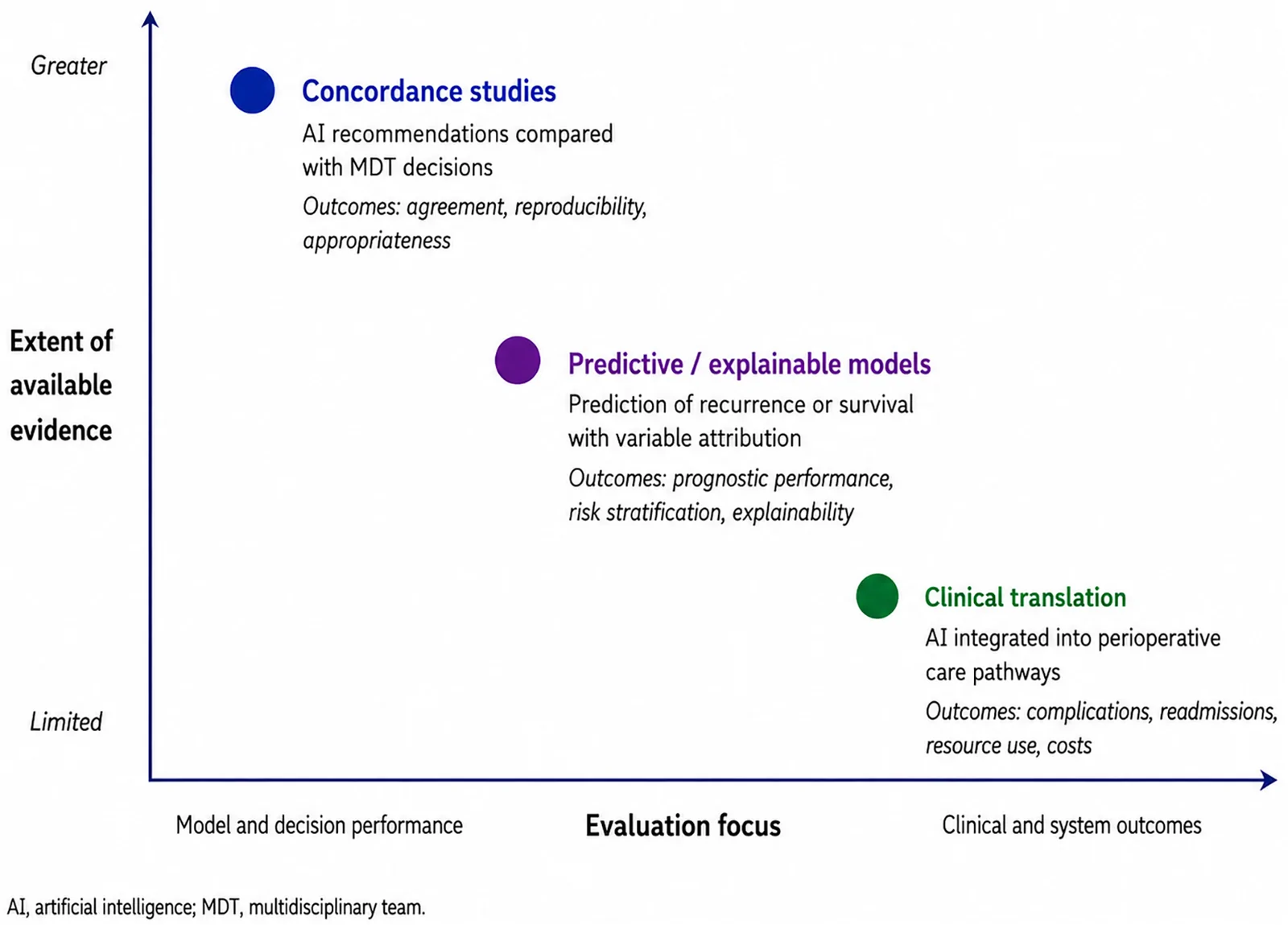
Qualitative evidence landscape of AI-based clinical decision support relevant to colorectal cancer multidisciplinary decision-making. Evidence domains are positioned qualitatively according to the evidence considered in this review and their evaluation focus. Positioning reflects the authors’ narrative synthesis rather than a formal evidence-grading or exhaustive evidence-mapping exercise. The CRC-specific clinical translation example reflects perioperative care rather than evidence of AI-supported MDT treatment decision-making.

### 4.1 Limitations and challenges of AI integration

The contemporary CRC-specific direct AI–MDT evidence base remains methodologically heterogeneous and dominated by retrospective, single-center LLM studies (28–36). Although two cohorts included 1,423 and 300 cases (28, 29), several others enrolled 15–179 patients and/or used clinically restricted cohorts (30–36). Evaluation methods and endpoints also vary substantially, including exact agreement, κ statistics, Likert ratings, output stability, and safety assessments (28–37), limiting cross-study comparison and conclusions regarding clinical effectiveness. A major translational gap in the contemporary evidence reviewed here is the scarcity of prospective CRC-specific evaluation of AI integrated into MDT deliberation and its effect on decisions. The perioperative study provides clinical deployment evidence outside direct MDT decision-making but was non-randomized and could not separate effects of the decision-support tool from standardized treatment bundles (40).

Available evidence suggests that disease stage and clinical severity may influence concordance, but the relationship is inconsistent. Lower agreement was reported in metastatic, stage IV, or recurrent disease in several studies (28–30), whereas T or N stage was not consistently associated with concordance, and high agreement or higher concordance scores were also observed in selected advanced or node-positive cohorts (28, 31, 33, 34). Other studies reported lower agreement in older patients or those with poorer performance status, differences according to pathological risk burden, and marked changes after explicit provision of resectability information, while model configuration also affected the results (29, 31, 32, 35). Some of these subgroup findings were exploratory or descriptive and should therefore be interpreted cautiously (29, 30). Taken together, concordance appears to reflect the interaction of disease burden, age, performance status, treatment context, information completeness, and model configuration rather than TNM stage alone.

Beyond these general limitations, LLM-based systems are the most frequently studied approach and present challenges specific to their mode of operation (28–37). Their performance appears to depend heavily on the quality, completeness, and structure of the input data. This dependence is reflected in the greater frequency of unstable outputs among cases with incomplete staging information (28) and in changes in recommendations when clinically decisive information, such as resectability, was explicitly added (35). Model configuration and unequal access to external guideline information may also influence observed performance differences between models (29). Additionally, reproducibility remains inconsistent across studies, ranging from complete within-model consistency under fixed conditions to unstable or only moderately consistent repeated outputs (28, 35, 37). Reproducibility also depends on transparent reporting of the model and version, access date, prompting strategy, repeated query protocol, browsing or retrieval access, and sampling parameters such as temperature where configurable. Another important concern is that LLMs may generate recommendations supported by lower-level or preclinical evidence, which may broaden therapeutic options but also increase the risk of clinically inappropriate or weakly supported suggestions (37).

Achieving adequate transparency and explainability remains an important challenge across AI approaches. For LLMs, it can be difficult to determine how specific recommendations are generated and how different inputs are weighted (20, 37). Explainability methods have also been incorporated into colorectal cancer predictive models, one of which was deployed as a web-based system on a hospital’s internal network; however, both studies were retrospective, and whether such explanations improve clinician understanding, clinical decision-making, or patient outcomes has not been prospectively assessed (16, 17). AI integration into MDT practice raises practical, ethical, and medico-legal concerns, particularly regarding accountability when recommendations differ from standard practice or result in adverse outcomes (41). It also requires careful attention to data privacy, governance, and secure handling of sensitive patient information (20). These challenges, together with the need for transparency and clinical responsibility, indicate that AI should currently be regarded as a support tool rather than a replacement for expert multidisciplinary judgment (20, 37, 41).

### 4.2 Future directions

Future research should focus on prospective, multicenter studies evaluating AI incorporated into colorectal cancer MDT workflows. Beyond concordance or predictive performance, studies should assess the independent appropriateness or safety of AI recommendations, effects on MDT decisions and workflow, and patient or system outcomes (16, 17, 31, 39, 40). Standardized evaluation frameworks are essential to allow meaningful comparison across studies, given the current heterogeneity in study design, endpoints, and reporting (28–40). Furthermore, broader validation across different institutions and healthcare systems will be required to determine the generalizability and clinical robustness of these tools.

Based on the evidence reviewed, we consider the most realistic near-term role of AI in this field to be as a supervised adjunct rather than an autonomous decision-maker (20, 37, 41). In clinical practice, its value may lie in structuring complex case information, drawing attention to missing or clinically decisive variables, and providing preliminary risk estimates or management options for multidisciplinary review. Concordance with MDT recommendations should not be regarded as the sole measure of clinical value, because discordance may reflect model limitations, incomplete clinical information, or legitimate variation in expert and institutional practice (8, 24, 28, 35). Hybrid models combining AI-supported analysis with expert multidisciplinary review may therefore offer the most balanced approach, potentially improving efficiency while preserving clinical judgment and accountability. Future systems should aim to integrate multimodal data, including imaging, pathology, molecular profiling, and clinical variables, within a structured and standardized framework, to provide more comprehensive and context-aware support. At the same time, wider implementation will require improvements in transparency, reproducibility and evidence attribution (20, 37). Finally, clear ethical and regulatory frameworks will be necessary to ensure that AI supports, rather than replaces, expert decision-making (20, 41).

## 5 Conclusion

Artificial intelligence is emerging as a promising adjunct in multidisciplinary decision-making for colorectal cancer, although reported concordance with MDT recommendations varies across clinical settings, models, and evaluation methods. However, the available literature remains limited by predominantly retrospective designs, many small or selected cohorts, heterogeneous endpoints, and sparse CRC-specific prospective evaluation of AI in MDT decision-making. At present, AI should be regarded as a supervised support tool rather than a replacement for expert multidisciplinary judgment. Further prospective, multicenter studies are required to determine whether integration into MDT workflows can improve MDT decision quality, workflow performance, or patient outcomes.
